# Effects of an Individualized vs. Standardized Vitamin D Supplementation on the 25(OH)D Level in Athletes

**DOI:** 10.3390/nu15224747

**Published:** 2023-11-10

**Authors:** Chiara Tuma, Arne Schick, Nele Pommerening, Hans Braun, Mario Thevis

**Affiliations:** 1 Institute of Biochemistry/Center of Preventive Doping Research, German Sport University Cologne, 50933 Cologne, Germanythevis@dshs-koeln.de (M.T.); 2 German Research Centre of Elite Sports (Momentum), German Sport University Cologne, 50933 Cologne, Germany; 3 European Monitoring Center for Emerging Doping Agents, 50933 Cologne, Germany

**Keywords:** vitamin D, sports nutrition, personalized nutrition, microsampling, individualization

## Abstract

Vitamin D is crucial to the health and performance of athletes. Although the exact vitamin D requirements for athletes have not been established, maintaining a 25(OH)D level of at least 40 ng/mL is considered beneficial. This randomized controlled intervention study aimed to determine whether an individual loading dose formula for vitamin D supplementation is more effective than standardized supplementation and suitable enough for athletes to meet a target value of 40 ng/mL. In a 10-week supplementation study conducted during the winter months in Germany, 90 athletes with insufficient vitamin D levels (25(OH)D < 30 ng/mL) were randomly assigned to receive either a universal dose of 2000 IU/day of vitamin D or a loading dose of 4000 IU/day, followed by a maintenance dose of 1000 IU/day. The total 25(OH)D concentration was measured from dried blood spots at three time points: at baseline, at the computed date of 40 ng/mL, and after the 10-week period. Additionally, a vitamin-D-specific questionnaire was issued. On the day when 25(OH)D blood concentrations of 40 ng/mL were calculated to prevail, the individualized group had a significantly higher 25(OH)D level than the standardized group (41.1 ± 10.9 ng/mL vs. 32.5 ± 6.4 ng/mL, *p* < 0.001). This study demonstrated that the examined formula is suitable enough for athletes to achieve a 25(OH)D concentration of 40 ng/mL. This indicates that a personalized approach is more effective than a one-size-fits-all approach in restoring adequate vitamin D levels in athletes.

## 1. Introduction

Vitamin D has hormone-like effects that exert its actions ubiquitously in the human body [1]. This is why it is not only indispensable for general health but also plays a role in athletes’ performance. Generally, there are two forms of vitamin D: vitamin D2 (ergocalciferol) and vitamin D3 (cholecalciferol).

Vitamin D is crucial for athletes whose bones may be more strained than those of the general population because of its main impact on bone metabolism by modulating calcium resorption from the bone [1]. Recent studies have shown the considerable impact of vitamin D on the immune system and muscle function [2,3,4]. The significance of the impact of vitamin D on inflammatory markers is noteworthy for athletes, given the fact that extreme endurance training can cause oxidative stress, muscle damage, and inflammation [5,6,7,8,9]. Serum 25(OH)D levels are negatively correlated with circulatory inflammatory markers, peak power, and skeletal muscle strength [10]. Notably, when Interleukin-6 (IL-6) levels are increased during exercise, as it is acutely released from working muscle fibers with increased exercise duration, intensity, and muscle glycogen depletion, vitamin D may exert positive effects [11].

Given its active role in muscle function, the potential of vitamin D to enhance athletic performance is a topic of much debate [12,13]. It has been suggested that elite athletes should aim for a 25(OH)D concentration of 40 ng/mL, which is higher than the standard 30 ng/mL for non-athletes [14,15,16,17,18]. Vitamin D insufficiency is typically characterized by a 25-hydroxyvitamin D (25(OH)D) concentration < 30 ng/mL; whereas, vitamin D deficiency is defined as a 25(OH)D concentration < 20 ng/mL. Recent studies have indicated that the prevalence of vitamin D insufficiency and deficiency in various sports populations is up to 73% and 62%, respectively [16,19,20].

To date, no athlete-specific recommendations have been established for vitamin D intake or supplementation. Athletes at a higher risk of vitamin D inadequacy may benefit from vitamin D supplementation guidelines. These include indoor athletes, athletes living at a latitude of 50° N or higher, and athletes with training regimens in the early mornings or evenings [21,22,23].

Several investigations have been undertaken to determine the optimal vitamin D supplementation regimens for correcting vitamin D status when endogenous synthesis is impaired. However, none of these studies provided specific guidelines applicable to athletes’ daily lives. Reference values for vitamin D intake for adults in Northern Europe (latitudes above 40° N) range from 200 to 800 IU per day [24] and the recommended daily intake (RDI) for adults, as established by various institutions and sports organizations, varies from 400 to 2000 IU per day [25]. According to Holick et al. [26], doses of at least 1500–2000 IU/day are required to raise the blood level of 25(OH)D above 30 ng/mL. Conversely, Barger-Lux et al. [27] proposed that an average of 3000–5000 IU/day of cholecalciferol is used daily by the body, without considering the increased vitamin D requirements for athletes with high levels of physical activity.

In addition, the tolerable upper intake level (UL) of vitamin D exhibits a range of discrepancies among the various sources. According to the EFSA Panel on Dietetic Products, Nutrition, and Allergies (NDA) [28], the UL is set at 4000 IU per day; whereas, Holick et al. [26] proposed an UL of 10,000 IU per day.

As standardized recommendations for vitamin D intake do not exist, and due to many factors influencing vitamin D status, a more individualized supplementation strategy for vitamin D status optimization should be targeted. A personalized approach to correct insufficient vitamin D status could be based on body weight and the 25(OH)D baseline concentration. In 2010, Groningen et al. published a formula to correct individual vitamin D deficiencies [29]. This formula establishes the total dose required to attain a sufficient vitamin D level of 30 ng/mL. However, the optimal method for dividing the total dose into daily doses remains unclear and, to the best of our knowledge, no data have been published on whether this formula applies to athletes.

The objective of this study was to determine the applicability of Groningen’s formula in athletes. To achieve this, this study compared the effectiveness of individualized vitamin D supplementation versus standardized supplementation in correcting inadequate 25(OH)D concentrations enough to reach a level of 40 ng/mL. Therefore, for a 10-week intervention period, athletes with an insufficient vitamin D status (25(OH)D < 30 ng/mL) were administered either a loading dose followed by a maintenance dose or a standardized dose throughout the study period.

## 2. Materials and Methods

### 2.1. Participants

Ninety athletes between 18 and 50 years of age who exercised at least five hours per week were included. All participants lived in Germany (48–55° N) for the entire duration of this study. To avoid endogenous vitamin D synthesis, participants were instructed not to spend time in a region with a UV index greater than 3. Additionally, participants already supplemented with vitamin D2 or D3 were excluded from the trial.

All procedures followed in this study involving human participants were in line with the ethical standards of the institutional research committee and with the 1964 Helsinki Declaration and its subsequent amendments. Prior to this study, ethical approval was obtained from the local ethics committee of the German Sport University Cologne (#232/2022) and written consent was obtained from all participants.

### 2.2. Study Design

The study duration was 10 weeks. This study was conducted during the winter months, between January and April, in Germany. The UV index was continuously below 3 for the entire 10 weeks. The daily UV index was provided by the Federal Office for Radiation Protection (BfS) and was measured using an array spectroradiometer.

The first blood sample of each athlete was obtained in a one-week period before the start of this study. Afterward, the participants were randomized using matched-pair randomization based on baseline 25(OH)D concentrations. Athletes with established vitamin D inadequacy (25(OH)D3 level < 30 ng/mL) were randomly assigned to one of three groups: individualized supplementation (INDV), standardized supplementation (STAD), and a control group (CON). Athletes with a sufficient vitamin D status (25(OH)D3 level ≥ 30 ng/mL) (SUF) did not receive any supplementation but were also monitored until the end of this study.

Vitamin D3 supplements were obtained from RheinNutrition (R(h)ein Nutrition UG & Co. KG, Cologne, Germany). All capsules provided were from the same batch and comprised cellulose capsules containing 1000 IU of cholecalciferol. The manufacturer reported a maximum allowable variation in vitamin D content of 5%.

The individualized supplementation regimen was based on the formula described by Groningen et al. [29], which was adapted from the target concentration of 75 to 100 nmol/L (30 to 40 ng/mL).

$$total\;dose\left(IU\right)=40\times \left(100-serum\;25\left(OH\right)D\right)\times body\;weight$$

**Figure 1:**
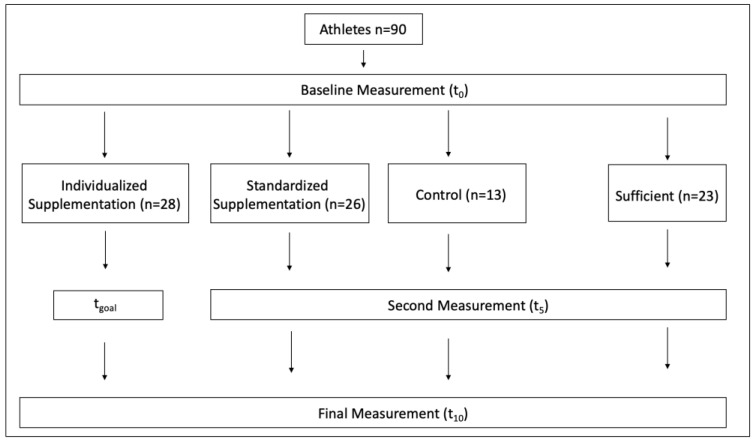
Overview of the different measurement time points for the INDV, STAD, CON, and SUF groups.

The resulting total dose was divided into daily doses of 4000 IU, corresponding to four capsules daily. After the calculated time provided to reach the goal status of 40 ng/mL (t_goal_), participants received 1000 IU daily for maintenance until the end of the study period. Standardized supplementation consisted of 2000 IU, corresponding to two capsules, daily. All participants were instructed to take their capsules once a day, together with a meal. The second blood sample from the INDV group was taken maximally 48 h after the individually calculated time point when a 25(OH)D concentration of 40 ng/mL was reached (t_goal_), which was calculated beforehand. The second set of blood samples from the STAD, CON, and SUF groups were collected after 5 weeks. At the end of this study, final blood samples from all groups were collected 10 weeks after the commencement of supplementation. This measurement time was standardized to 24–48 h after the last supplementation day. Figure 1 shows the schedule with the measurement points for all of the groups.

### 2.3. Analytical Parameters

Vitamin D status was assessed by measuring the sum of 25(OH)D2 and 25(OH)D3 serum concentrations, hereafter referred to as total 25(OH)D. The total 25(OH)D was quantified from 20 μL Mitra tips using volumetric absorptive microsampling technology (VAMS^®^) (Neoteryx, CA, USA) [30]. Mitra tips consist of an absorptive polymetric tip attached to a plastic handler that is designed to take up a fixed volume of blood of 20 μL.

Capillary blood samples were collected through a finger prick using Microlet^®^ lancets (Bayer Health Care, Leverkusen, Germany). The VAMS^®^ sample preparation was conducted by a trained researcher, in accordance with the manufacturer’s instructions at the Institute for Biochemistry of the German Sports University Cologne, in a manner that ensured the highest level of accuracy and precision. After preparation, the samples were dried for at least three hours at room temperature. Subsequently, the VAMS^®^ samples were stored individually in Ziploc plastic bags with a desiccant (silica gel) at −80°C until analysis. Samples were analyzed in duplicate using a validated UHPLC-HRMS method [30]. The current study used VAMS^®^ samples instead of serum for 25(OH)D quantification. A primary distinction between the two matrices is that VAMS samples contain whole blood while serum is derived from centrifuged blood. To ensure accurate comparisons between the VAMS^®^ and serum values, independently certified serum quality controls from Chromsystems (Munich, Germany) were utilized for correction. Standardized VAMS^®^ samples with a hematocrit of 0.40 were produced by combining washed red blood cells with certified serum controls at two known 25(OH)D levels.

### 2.4. Questionnaire

In addition to the vitamin D status assessment, participants completed a vitamin-D-specific questionnaire at three time points throughout the study period: at the beginning and after 5 and 10 weeks, respectively. The questionnaire used in this study was based on the validated vitamin-D-specific food frequency and lifestyle questionnaire (FFLQ) by Larson-Meyer et al. [31]. It was modified for athletes by adding sport-specific questions and was adapted for German food choices. Furthermore, individual skin types after Fitzpatrick were determined by considering eye and hair color as well as individual responses to sunlight [32].

### 2.5. Statistical Analysis

A power analysis was performed before the commencement of this study using G*Power [33]. To determine whether there is a significant difference in 25(OH)D concentration in response to either an individualized or standardized supplementation strategy when compared to two control groups, and to achieve a power (1-β) of 80% and a significance level (α) of 0.05, a minimum of 76 participants, in total, were required. This calculation assumed a large effect size of f = 0.40.

All data were analyzed using intent-to-treat (ITT) analysis. Results are presented as mean ± standard deviation (M ± SD). Outliers were analyzed using box plots and defined as values beyond the 1.5-fold interquartile range (IQ).

The normality and homogeneity of variances were assessed using Shapiro–Wilk’s and Levene’s tests. One-way ANOVA with Tukey’s post hoc analysis was conducted to analyze the differences between the four groups at a specific measurement time point. Repeated-measures ANOVA with Bonferroni-adjusted post hoc analysis was conducted to detect differences in 25(OH)D concentrations within the groups over time.

Repeated-measures analysis of covariance (ANCOVA) in conjunction with a Bonferroni post hoc test was performed to evaluate the differences in 25(OH)D concentrations among the groups over time, controlling for confounding variables, such as BMI, training volume, and age.

The Pearson correlation coefficient was calculated to identify correlations between 25(OH)D concentrations and possible influencing factors, such as BMI, training and competition volume, or nutritive vitamin D uptake.

The Eta-squared statistic (η^2^) was used to calculate the effect size. Statistical significance was set at *p* value < 0.05. Data were analyzed using SPSS Statistics version 29 (SPSS, Chicago, IL, USA).

## 3. Results

**Figure 2:**
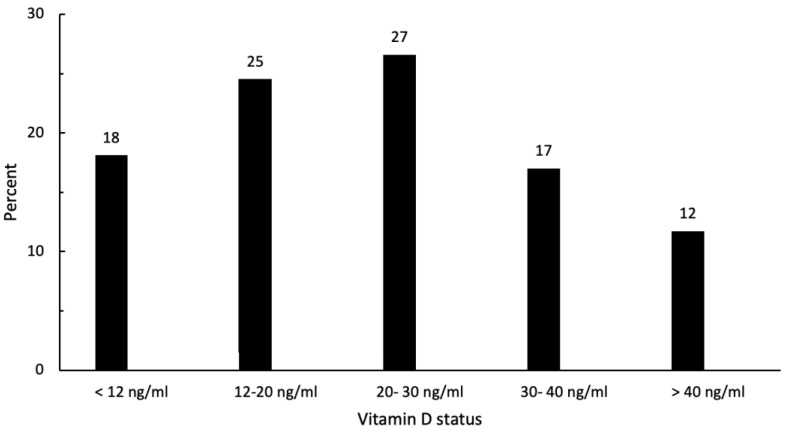
Categorized distribution of 25(OH)D concentrations (n = 90) at baseline.

A total of 90 athletes with an average training volume of 7.4 ± 4.1 h per week, an average age of 26 ± 5 years, and an average BMI of 23 ± 3 were recruited for the first vitamin D measurement. At baseline, 67 athletes (74%) had an insufficient vitamin D status (25(OH)D level < 30 ng/mL); whereas, a total of 23 (26%) had a sufficient vitamin D status (25(OH)D level ≥ 30 ng/mL). Figure 2 shows the classified distribution of the baseline 25(OH)D concentrations among the 90 athletes. The baseline characteristics of the participants are shown in Table 1.

One-way ANOVA revealed no significant difference in the mean baseline 25(OH)D concentrations between the INDV, STAD, and CON groups (17.5 ± 7.6 vs. 17.0 ± 7.2 vs. 21.9 ± 6.3 ng/mL). Compared to these, the baseline 25(OH)D concentration of the SUF group was significantly higher at 40.5 ± 8.2 ng/mL (*p* < 0.001, η^2^ = 0.65). The 25(OH)D concentrations of all four groups at all time points are shown in Table 2.

Within the entire 10-week study protocol, the mean 25(OH)D level significantly increased by 20.4 ng/mL in the INDV group (*p*<0.001, η^2^ = 0.73) and 21.1 ng/mL in the STAD group (*p* < 0.001, η^2^ = 0.78). In the INDV group, the 25(OH)D concentration significantly increased from the baseline to the pre-calculated reaching point (23.6 ng/mL, *p* < 0.001). From the pre-calculated reaching point to the final measurement, 25(OH)D declined by 3.2 ng/mL. In the STAD group, the 25(OH)D concentration increased steadily from the baseline to the final measurement: after 5 weeks, the 25(OH)D concentration significantly increased from the baseline (+15.5 ng/mL, *p* < 0.001). Within the second 5-week period, the 25(OH)D concentration increased further from 32.5 ± 6.4 to 38.1 ± 6.8 ng/mL (*p* < 0.01).

In total, 50% of the INDV study group volunteers reached the target concentration of 40 ng/mL at the pre-calculated time point and 92% reached 30 ng/mL. However, after the total 10-week supplementation protocol, only 30% showed a 25(OH)D concentration of at least 40 ng/mL and 78% showed a 25(OH)D concentration of 30 ng/mL or more.

In the STAD group, 17% showed a vitamin D level of 40 ng/mL or higher after 5 weeks and 38% after 10 weeks. On average, 41 ± 12 days elapsed until the insufficient vitamin D status was corrected in the INDV group.

In the CON group, 25(OH)D concentration decreased over the 10-week study period (−3.8 ng/mL). The 25(OH)D concentration significantly decreased in the first 5 weeks from 21.9 ± 6.3 to 17.7 ± 6.2 ng/mL (*p* = 0.05, η^2^ = 0.48) but did not decrease further in the last 5 weeks (17.7 ± 6.2 vs. 18.1 ± 6.3 ng/mL).

In the SUF group, 25(OH)D significantly decreased in the first 5 weeks (−8.9 ng/mL, *p* < 0.005, η^2^ = 0.46), as well as in the total 10-week study period (−8.7 ng/mL, *p* < 0.01). Similar to the CON group, the mean 25(OH)D concentration did not further decline in the last five weeks (31.6 vs. 31.8 ng/mL, *p* > 0.05). There was no significant difference in the decrease in 25(OH)D concentration over the 10 weeks between the CON and SUF groups (−3.8 vs. −8.7 ng/mL, *p* = 0.13).

Repeated-measures ANCOVA in the supplementation groups revealed a significant difference in 25(OH)D concentration over time, independent of the group, when controlling for training volume (F(2,60) =5.24, *p* < 0.01).

There was a negative correlation between the weekly average training volume and the increase in 25(OH)D during the 10-week supplementation protocol (r = −0.34, *p* < 0.05), and a strong negative correlation between the baseline concentration and the rise in 25(OH)D during the 10-week supplementation protocol (r = −0.54, *p* < 0.001).

In the CON and SUF groups, there was a negative correlation between the baseline concentration and the decline in 25(OH)D during the 10-week observation period (r = −0.45, *p* < 0.05). There were no other significant correlations between vitamin D status and possible influencing factors (Table 3).

Nutritional vitamin D intake was calculated using the means of the different chosen foods available in Germany. The average daily vitamin D uptake was 3.6 ± 2.3 μg/day.

Dietary vitamin D intake did not affect the total 25(OH)D concentration (r = −0.23, *p* > 0.05).

## 4. Discussion

The objective of this study was to compare the efficacy of individualized and standardized vitamin D supplement regimens for correcting insufficient 25(OH)D levels, as well as to determine the applicability of Groningen’s formula to athletes with possibly elevated requirements for vitamin D.

The current supplementation study involved a cohort of young, healthy German athletes who were at an elevated risk of vitamin D deficiency during the winter months, which may negatively impact their athletic performance and health status. This study’s findings reiterate the limited impact of nutritional vitamin D intake on 25(OH)D levels, underscoring the challenges associated with achieving a sufficient vitamin D status through dietary means. Instead, this study suggests that vitamin D supplementation may be necessary during periods when vitamin D synthesis is limited.

During the middle of winter, only 26% of the examined athletes had a sufficient vitamin D status (25(OH)D > 30 ng/mL). This observation is consistent with other studies conducted at similar latitudes that reported vitamin D insufficiency rates ranging from 40 to 60% [19,23,34].

The results provide evidence that the formula established by Groningen et al. [29] is also applicable to athletes with higher vitamin D demands. Replacing the target concentration of 30 ng/mL with 40 ng/mL in the formula resulted in a mean 25(OH)D concentration of 41.1 ± 10.9 ng/mL at the pre-calculated time point.

Although the calculated loading dose using Groningen’s formula delivered good results, when split up into daily doses of 4000 IU, the mean 25(OH)D concentration decreased afterward, during the maintenance phase (37.9 ± 10.3 ng/mL). This indicated that the maintenance dose of 1000 IU/day was not high enough to ensure the target concentration of 40 ng/mL in athletes.

**Figure 3:**
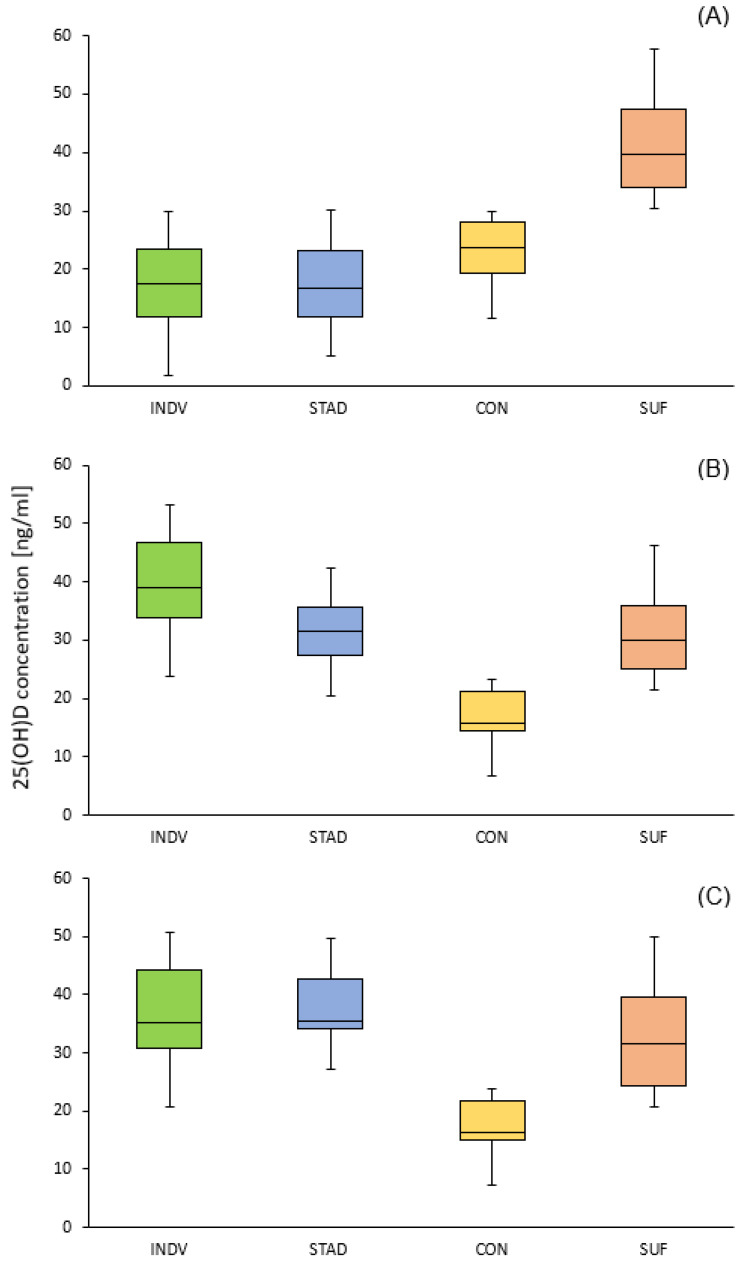
25(OH)D concentrations at baseline (**A**), at the second measurement (**B**), and at the final measurement (**C**) for all groups (INDV, STAD, CON, and SUF).

As shown in Figure 3, the individual 25(OH)D concentrations from the INDV group declined in most cases (57%) from the second to the third measurement. In contrast, in the STAD group (84%), 25(OH)D concentration steadily increased throughout the 10 weeks.

This result is consistent with the findings of Backx et al. [34], who showed that the average 25(OH)D concentration decreased from 47.6 to 30.4 ng/mL in the winter months when 1100 IU of cholecalciferol per day was administered. Ogan and Pritchett [16] proposed that athletes with vitamin D insufficiency should consume 5000 IU of cholecalciferol daily in the winter months to reach a level of 40 ng/mL. As a maintenance dose, they proposed 1000–2000 IU/day. Our results support these recommendations; however, a maintenance dose > 1000 IU/day may be required in some cases to maintain an upright status of 40 ng/mL. Therefore, when establishing vitamin D supplementation strategies, dosages to maintain a certain 25(OH)D concentration must be clearly differentiated from dosages to correct an insufficient or deficient status.

In the INDV group, the mean 25(OH)D levels reached 41.1 ± 10.9 ng/mL at the calculated time point; on an individual basis, only 50% reached the target concentration of 40 ng/mL or more. This may be due to inter-individual differences. A closer look at the differences in the characteristics of those who reached 40 ng/mL and those who did not shows that the latter had a significantly higher weight than those who reached 40 ng/mL or more at the calculated time point (95%CI [−0.21, 18.66]), 68.5 vs. 77.7 kg, *p* < 0.05, d = 0.85). Unfortunately, fat mass and fat-free mass were not measured in this study.

The findings of this study are consistent with those of previous research, which has demonstrated an inverse relationship between the serum 25(OH)D levels achieved using a fixed dose of oral cholecalciferol and both body weight and BMI. The mechanism underlying this relationship is believed to be a greater distribution volume resulting from increased body fat content [35,36]. However, the loading dose in the formula considered body weight; in the present study, this adjustment seemed insufficient for athletes.

To date, there has not been a consensus on tolerable upper intake level (UL): actual guidelines vary between 4000 [28] and 10.000 IU [26] daily. The current investigation considered an upper daily intake limit of 4000 IU/day, which can be considered moderate in this context.

Although supplementing with higher doses may expedite the correction of an inadequate vitamin D status, the administration of doses exceeding these ULs should be carefully considered as higher doses of supplementation may pose a heightened risk of overdosing, especially when athletes are not supervised individually. According to a study conducted by Arabi et al. [37], it has been observed that non-physiological high doses of vitamin D can result in an upregulation of countervailing factors, leading to a lower synthesis of the biologically active hormone 1,25-dihydroxyvitamin D. As a result of prolonged exposure to high doses of vitamin D, it may ultimately lead to the downregulation of cellular activation and immunity factors [38]. Conversely, a moderate daily dose of vitamin D3 has superior intracellular effects; however, it must be taken more frequently because of its 20-h half-life [39,40,41].

While the requirement for vitamin D in athletes has not been definitively established, several investigations have indicated a significant correlation between vitamin D insufficiency and athletic performance. These include power and strength parameters, such as vertical jump, muscle power, and mean power output in combat sports [42,43,44,45], as well as endurance parameters, such as decline in VO_2_max or submaximal performance on a treadmill [46,47,48,49]. Previous studies have shown a strong correlation between the prevalence of lower extremity muscle strains, core muscle injuries, and low vitamin D status [50,51]. In athletes, the prevalence of stress fractures, such as leg and foot bone injuries, among musculoskeletal injuries seems to be significantly higher in athletes with vitamin D insufficiency (*p* < 0.001) [52,53,54]. Mieszkowski et al. [55] even showed an anti-inflammatory effect of vitamin D supplementation on ultramarathon-induced inflammation in athletes with mean 25(OH)D concentrations < 30 ng/mL.

Regular assessments of vitamin D status throughout the year are necessary to address the high prevalence of vitamin D insufficiency in athletes. In sports practice, the feasibility of such regular measurements can be increased by implementing volumetric absorptive microsampling technology (VAMS^®^) into athletes’ daily lives.

One of the strengths of this study is the high individuality of the supplementation strategies analyzed. Each athlete in the individualized supplementation group was measured at the individually calculated time point of reaching a 25(OH)D concentration of 40 ng/mL. This was mainly achieved using a microsampling technique for blood collection, such as VAMS^®^. To the best of our knowledge, this is the first study to use VAMS^®^ sampling for vitamin D status measurement in athletes. Compared to classical venipuncture, VAMS^®^ offers several benefits for sample collection, transport, and storage. First, the sample collection is highly independent for athletes and researchers. Because there is no need for medical staff or to see a doctor, athletes can collect samples themselves at any place, for example, at home or at training sites. Afterward, the samples can be easily sent by mail to the laboratory and stored frozen for several months until analysis.

Moreover, only a few microliters of capillary blood are needed instead of needing to collect a few milliliters. Therefore, in practice, the frequency of performing venipuncture is a limiting factor and, thus, favors the use of microsampling in frequent sample collection. Compared to dried blood spots (DBS), autonomous sample collection is facilitated and accelerated in VAMS^®^ because blood does not need to be transferred from the fingertip to the collection device. Instead, the Mitra^®^ device can be applied directly to the fingertip. This not only reduces imprecision but also improves the feasibility when samples are taken without supervision. To ensure high quality in practice, athletes must be properly introduced to autonomous VAMS^®^ sample collection. Therefore, clear instructions for untrained users are essential. Van Uytfanghe et al. [56] reported that watching an online instruction video and stressing the importance of correct sampling were sufficient enough to provide a high sample quality in self-sampling at home.

When analyzing whole blood samples, it is important to consider the influence of the hematocrit. Denniff and Spooner [57] demonstrated that VAMS^®^ sampling is effective in overcoming hematocrit bias, which can affect the accuracy of classical DBS [58,59]. They found that the difference in the sampled blood volume was 5% over the hematocrit range of 20–70%. In comparison, variations in the blood volume of DBS subpunches were 30% over the same hematocrit range [60]. Jensen et al. [61] confirmed these findings, reporting varying 25(OH)D3 concentrations in 3.2 mm diameter DBS punches. They found a bias ranging from +16% to −22% over a hematocrit range from 30% to 60%, when normalized to a hematocrit of 45%, in healthy subjects. However, the impact of hematocrit on the assay must be evaluated accordingly. Newman et al. [62] stated that the hematocrit of healthy adults mostly varies between 40% and 60%. Additionally, Ackermans et al. [63] found that 25(OH)D concentrations were independent of the hematocrit in a healthy range of 30–50%. Based on the assumption that an exact volume of 20 μL of capillary blood was absorbed by each Mitra^®^ device, the hematocrit bias was excluded from the developed assay when applied to healthy subjects.

Finally, it is imperative to acknowledge the limitations of this study. The generalizability of these results is subject to certain limitations. For instance, athletes with an average training volume of 7.4 ± 4.1 h per week were included in this study. Elite athletes with considerably higher training volumes may have produced slightly different results. Moreover, the current research was not specifically designed for specific sports disciplines; rather, it included athletes from different kinds of indoor and outdoor sports. Another limitation is the exact calculation of individualized supplementation. The baseline 25(OH)D concentration was assessed within a period of one week before the start of the 10-week study period. Therefore, the duration of the loading dose did not consider this one-week period in which vitamin D status could have declined further until supplementation started. This discrepancy may have influenced the efficiency of the application of the formula and may explain why not all athletes reached the goal status at the pre-calculated time points. It is also important to note that the samples were collected within 24 h and, in some cases, up to 48 h, after the pre-calculated threshold of 40 ng/mL was reached. Participants who received their final loading dose on the day of the second measurement may not have fully absorbed and metabolized it at the time of testing. It is worth mentioning that Chen et al. [64] found that the peak level of 25(OH)D occurred three days after vitamin D supplementation. However, that study administered a single dose of 300,000 IU, which cannot be directly compared with the doses used in the present study.

Nonetheless, these issues need to be considered in the practical application of the formula because, in practice, it will be rare for the results of vitamin D status measurements to be available on the same day.

Finally, this study did not consider the severity of vitamin D insufficiency. The supplementation provided to the INDV group was standardized to 4000 IU daily, which meant that athletes with severe deficiency did not receive a higher daily loading dose than athletes with mild insufficiency. As a result, these athletes had a much longer period with a deficient vitamin D status. It is appropriate to consider the potential utility of customizing the initial loading dose based on the degree of vitamin D insufficiency, such as increasing the daily dose up to 10,000 IU in a closely monitored environment.

Although this study has some limitations, it emphasizes the importance of vitamin D supplementation strategies for athletes, especially when endogenous synthesis is impaired, as these requirements cannot be provided solely from nutrition. To prevent a possible decline in athletic performance and long failure times due to illness or injuries, athletes need to be informed and adequately supported regarding vitamin D. It has been previously stated that an individualized approach is warranted when correcting vitamin D deficiency [14]. The approach used in this study, using independent VAMS^®^ sampling for vitamin D status assessment followed by an individualized supplementation strategy, is an example of how this individual support could be implemented in sports practice. In a controlled setting, the loading phase dosage could also be adapted to the severity of vitamin D deficiency to correct 25(OH)D concentrations faster and even more efficiently.

A potential strategy for incorporating vitamin D measurement and supplementation in sports practice could involve athletes measuring their 25(OH)D levels at the end of summer and during the winter months using a VAMS^®^ device, followed by the development of a personalized supplementation strategy consisting of both loading and maintenance doses. To further elucidate the effects of the formulation, future investigations should consider exploring various dosage forms for both the loading and maintenance phases. Further research is required to establish the connection between sports volume in elite athletes and vitamin D status, as well as to determine the optimal 25(OH)D concentrations and consistent supplementation strategies for individual sports.

## 5. Conclusions

Vitamin D supplementation plays a central role in covering vitamin D needs. Especially in winter months, when the UV index is too low for endogenous vitamin D synthesis, a carefully planned supplementation strategy is crucial. This research aimed to test the application of Groningens formula to increase the vitamin D needs of athletes and the establishment of an individual supplementation strategy for athletes. This study illustrated that an individualized approach for insufficient vitamin D status correction offers a more reliable and controlled strategy than a standardized approach. However, this also raises the question of the exact doses for the loading and maintenance phases. Daily doses of 4000 IU were effective in correcting insufficient vitamin D status. However, the daily maintenance dose for athletes should be higher than 1000 IU to maintain a 25(OH)D concentration of 40 ng/mL throughout the winter months. Based on these conclusions, practitioners should consider regular vitamin D monitoring throughout the winter months combined with a personalized vitamin D strategy. The methodology employed in this study, which entails independent and decentralized sample collection coupled with an individualized supplementation regimen, is well suited for application in sports settings. This approach could serve as a model for further investigations, such as the examination of the effects of varying loading and maintenance doses in different types of athletes.

## Figures and Tables

**Table 1:** Mean (±SD) subject characteristics of the individualized supplementation group (INDV), standardized supplementation group (STAD), control group (CON), and sufficient group (SUF).

	INDV (n = 28)	STAD (n = 26)	CON (n = 13)	SUF (n = 23)
Age (years)	25 (±5)	25 (±6)	26 (±4)	26 (±5)
Sex (M/F)	14/14	14/12	6/7	8/15
Height (cm)	176 (±9)	174 (±9)	175 (±12)	176 (±9)
Weight (kg)	74 (±11)	72 (±10)	71 (±13)	68 (±11)
Indoor/ Outdoor sport	23/5	18/8	6/7	10/13
Skin type after Fitzpatrick (2/3/4)	10/15/3	6/17/3	2/9/2	6/14/3
Training volume per week (h)	6.4 (±3.9)	7.3 (±4.4)	8.8 (±4.0)	8.0 (±4.1)
Competitions per month	0.6 (±1.9)	0.6 (±2.0)	0.2 (±0.6)	1.0 (±2.2)
Average nutritive vitamin D intake (μg/day)	3.5 (±2.5)	3.8 (±2.6)	4.1 (±2.3)	3.3 (±1.6)

**Table 2:** Total 25(OH)D concentrations (M ± SD) in ng/mL at three measurement points of the individualized supplementation group (INDV), standardized supplementation group (STAD), control group (CON), and sufficient group (SUF).

	INDV (n = 28)	STAD (n = 26)	CON (n = 13)	SUF (n = 23)
Baseline 25(OH)D concentration	17.5(±7.6) ^a^	17.0 (±7.2) ^a^	21.9 (±6.3) ^a^	40.5 (±8.4)
Second measurement ^#^	41.1 (±10.9) ^a,b,c,d^	32.5 (±6.4) ^c,d,e^	17.7 (±6.2) ^a,d^	31.6 (±10.7) ^d^
Final 25(OH)D concentration ^##^	37.9 (±10.3) ^c,d^	38.1 (±6.8) ^c,d^	18.1 (±6.3) ^a^	31.8 (±9.9) ^d^

Notes, ^#^ second measurement after 5 weeks for the STAD, CON, and SUF groups while the INDV measurement is based on a pre-calculated reaching point (after 41 ± 12 days); ^##^ third measurement for the INDV, STAD, CON, and SUF groups after 77 ± 12, 78 ± 13, 69 ± 4, and 75 ± 6 days; ^a^ significantly different from the CON group high at the same timepoint; ^b^ significantly different from the STAD group at the same timepoint; ^c^ significantly different from the CON group low at the same timepoint; ^d^ significantly different from the baseline concentration (t0); ^e^ significantly different from the final concentration (t10).

**Table 3:** Pearson correlation coefficients of possible influence factors in the supplementation groups and control groups.

	Supplementation Groups (INDV and STAD)		Control Groups (CON and SUF)	
	Baseline 25(OH)D concentration	Increase in 25(OH)D from baseline to final concentration (Δt_10_-t_0_)	Baseline 25(OH)D concentration	Decline in 25(OH)D from baseline to final concentration (Δt_10_-t_0_)
Difference in 25(OH)D from baseline to final measurement (Δt_10_-t_0_)	−0.54 **		−0.45 *	
Average training volume per week	0.19	−0.34 *	−0.18	0.01
Average competition volume per month	−0.12	0.01	0.17	0.03
Average nutritive vitamin D intake (μg/day)	0.03	−0.23	−0.09	0.01
BMI	−0.15	−0.001	−0.23	0.08

Notes * *p* < 0.05, ** *p* <0.001.

## Data Availability

Data will be made available on request.
